# Ecological suitability modeling for anthrax in the Kruger National Park, South Africa

**DOI:** 10.1371/journal.pone.0191704

**Published:** 2018-01-29

**Authors:** Pieter Johan Steenkamp, Henriette van Heerden, Ockert Louis van Schalkwyk

**Affiliations:** 1 University of Pretoria, Faculty of Veterinary Science, Department of Production Animal Studies, Onderstepoort, South Africa; 2 University of Pretoria, Faculty of Veterinary Science, Department of Veterinary Tropical Diseases, Onderstepoort, South Africa; ContraFect Corporation, UNITED STATES

## Abstract

The spores of the soil-borne bacterium, *Bacillus anthracis*, which causes anthrax are highly resistant to adverse environmental conditions. Under ideal conditions, anthrax spores can survive for many years in the soil. Anthrax is known to be endemic in the northern part of Kruger National Park (KNP) in South Africa (SA), with occasional epidemics spreading southward. The aim of this study was to identify and map areas that are ecologically suitable for the harboring of *B*. *anthracis* spores within the KNP. Anthrax surveillance data and selected environmental variables were used as inputs to the maximum entropy (Maxent) species distribution modeling method. Anthrax positive carcasses from 1988–2011 in KNP (n = 597) and a total of 40 environmental variables were used to predict and evaluate their relative contribution to suitability for anthrax occurrence in KNP. The environmental variables that contributed the most to the occurrence of anthrax were soil type, normalized difference vegetation index (NDVI) and precipitation. Apart from the endemic Pafuri region, several other areas within KNP were classified as ecologically suitable. The outputs of this study could guide future surveillance efforts to focus on predicted suitable areas for anthrax, since the KNP currently uses passive surveillance to detect anthrax outbreaks.

## Introduction

Anthrax is a rapidly fatal disease caused by the spore-forming bacterium *B*. *anthracis*. The disease can affect most species, but ruminants are particularly susceptible. Multiple host and environmental factors are thought to play a role in the transmission of anthrax. Many of Africa’s wildlife reserves experience cyclic anthrax outbreaks, one such area being the KNP in SA. However, little is known about the spatial ecology and epidemiology of anthrax [1]. The general thought is that *B*. *anthracis* is an obligate *in vivo* pathogen and that little propagation occurs in soil. Septicaemic infection with anthrax causes impaired clotting function [2]. When an animal succumbs to anthrax, the host’s impaired clotting ability results in blood draining into the soil from any orifice. If the environmental conditions are suitable, the bacterium will rapidly form spores once outside the host. Depending on the environmental conditions, these spores can survive for decades in the soil until infection of a suitable host takes place. Soil pH and soil calcium levels are considered the most important properties for spore survival and, therefore, endemicity of *B*. *anthracis* is associated with elevated calcium and neutral-to-alkaline soils [3–7]. Although these are considered the most important factors for the long-term survival of *B*. *anthracis* in the soil, other variables such as environmental temperature, rainfall, vegetation, presence of scavengers and mechanical vectors also play a vital role in the spread of the disease [8, 9].

Anthrax is considered an indigenous and integral part of the KNP ecosystem [2, 9]. The first confirmed case of anthrax in the KNP was in 1954, but it has been in the northern region of the Park for at least 200 years, as was proven by isolation of spores from archaeological bones dating back to 1700 ± 50 BC [10]. Anthrax outbreaks in KNP appear to have a cyclical pattern of roughly 10 years and are most often associated with a dry climatological spell after a couple of years of above average rainfall [11]. In an extensive wildlife reserve, such as the KNP, it is very difficult, if not impossible, to ensure immediate and proper disposal of anthrax-infected carcasses. Contaminated areas with anthrax spores in the soil and vegetation are thus constantly created by animals dying from the disease [12, 13]. Scavengers and predators may play a role in the dissemination of spores by the opening up and dispersal of carcasses, which could also have a dilution effect at the infected carcass site, but this remains speculative due to a lack of quantitative data. Spores can also be passed in scavengers’ feces to new sites or to water sources after feeding on infected carcasses and are disseminated by insects (blowflies in KNP) [8]. Water run-off [8] and infected carcasses [13] can contaminate the grazing that is in turn ingested by herbivores. Animals can stir up and ingest spores during the dry season when congregations occur around water points. Once an epidemic starts, the maintenance and spread of the disease are determined by biotic factors. Death from anthrax is per-acute to acute, although buffalo (*Syncerus caffer*) and eland (*Taurotragus oryx*) can travel great distances (~30 km) before a new infection locus will be established [11]. The dissemination of anthrax is still poorly researched since many of the assumptions are based on observation with no or little quantitative data available.

Kudu (*Tragelaphus strepsiceros*) are especially important in the spread of the disease since their numbers are positively correlated to food availability and therefore rainfall in the Park [14]. De Vos and Bryden [10], as well as Hugh-Jones and De Vos [8], indicated that an anthrax outbreak can be considered more likely when kudu numbers are high. This is explained by blow-flies that feed on body fluids of anthrax-infected carcasses and deposit their contaminated feces or regurgitated liquid on leaves of vegetation in the vicinity of the carcass [8, 11]. However, it has been shown that blow-flies can disperse up to 65 km from a carcass [15]. Browsing herbivores, such as kudu, that feed at a level where infected blow-fly regurgitated liquid are deposited on leaves (1–3 m) may then contract anthrax, starting a new cycle where the animal dies [11].

The identification of ecologically suitable areas for *B*. *anthracis* spores, and hence potential disease risk, is critical for the surveillance and management of the disease in wildlife, as wide scale immunization in wildlife remains untenable [16]. Passive surveillance is currently used to test potentially infected carcasses and monitor the extent of an outbreak (personal communication, State Veterinary Services, KNP, O. L. van Schalkwyk). Modeling of ecologically suitable areas for anthrax in the KNP may lead to a better understanding of anthrax ecology and epidemiology. Different, presence-only modeling approaches to predict the geographic distribution of *B*. *anthracis* have been used, such as Maxent [17] and GARP (genetic algorithm for rule-set prediction) [1, 18–20]. Tarkesh and Jetschke [21] compared correlative models including Worldclim, GARP and Maxent using the same input data and found Maxent achieved the best prediction. For the purpose of this study, ecologically suitable areas for anthrax spores were identified within KNP that are able to harbor the spores in sufficient quantities and for a period long enough to result in wildlife infection using the Maxent species distribution modeling method.

## Materials and methods

### Anthrax occurrence data

A database with a total of 597 confirmed positive anthrax cases in wildlife was constructed from historical records provided by the State Veterinary Services in Skukuza, KNP. These data were collected from 1988 to 2011. Maxent (version 3.3.3k, [22]) was used to determine ecological suitability for *B*. *anthracis* in the KNP. Maxent is a species distribution modelling technique that can be used with presence-only data and is considered equivalent to Poisson regression [22, 23]. To minimize model overfitting, the testing/training data set was created by classifying each pixel in the analysis framework of 18983 pixels (1 km^2^ resolution) as positive or negative, regardless of the number of cases in a positive pixel. A total of 219 positive pixels were identified (S1 Fig).

### Environmental variables

A total of forty environmental variables were selected (S1 Table). These included nineteen climatic variables from the Worldclim dataset (S2 Table) [24]. Six environmental variables including land type, landscape, soil data, lithology, geology and bore hole locations were provided by the KNP Scientific Services. From these six variables, a further three environmental variables (calcium (Ca), pH and distance to boreholes) were derived. Two layers—caventer and phventer were created from a dataset [25] with 370 soil sampling sites from KNP [26]. Inverse Distance Weighted (IDW) interpolation was used in ArcGIS (version 9.3.1) to derive continuous Ca and pH values. Ideally, specific measured values around KNP should be used, but this was not possible from the data available. Eight soil parameters (clay, cation exchange capacity, pH, potassium, calsium, magnesium, sodium, and phosphorus) were extracted from the SOil and TERrain (SOTER) digital database [27, 28]. Three distances to water layers (seasonal, ephemeral and permanent) were created, based on the hydrological index (HI) classification by Hannart and Hughes [29], which classifies rivers with a HI of 4.39–16.10 (class 1–4) as permanent, 16.11–37.81 (class 5) as seasonal and those with a HI larger than 37.81 as ephemeral (classes 6–9). A land cover layer was provided by the Peace Parks Foundation [30]. All environmental layers were rasterised, projected and resampled to a 1 km resolution before being clipped to the extent of the KNP. All datasets were prepared in ArcGIS. Sample selection bias was factored out by providing a target background layer [31, 32]. This layer was created by only selecting ranger sections of the KNP in which anthrax cases occurred over the study period. Since there are numerous ranger sections within the Park, this method provided an easy way to select the most appropriate target background layer. All the variables were evaluated for contribution importance using multiple regression and a jackknife procedure. The best subset of variables was selected based on outputs of the jackknife procedure, Maxent model surveyor (www.phycoweb.net/software), 95% gain confidence interval evaluation [33] and response curve interpretation [22]. Simple multiple regression was used to eliminate linearly correlated variables [34]. These selected variables were used for the second (final) model.

### Model parameters

A training dataset was used to build the model and an independent test dataset was used to measure how accurately the model can predict the points within this test set. A random test percentage option is also included in Maxent to indicate the percentage of data to use for testing and training. This dataset was divided into 75% training and 25% testing presence points (S1 Fig). Without this mutual exclusivity, the model would use the training data in testing, thus inflating model performance [35]. A total of 10 model runs were performed with convergence limit of 0.00001 and the maximum number of iterations set to 5000. A unique training and test dataset was created for each model run. The average value output map produced by Maxent was selected as the final output map for the model and is reported as the logistic value (continuous between zero and one, one being the highest suitability). In addition, an area under curve (AUC) measure was calculated as well as the true skill statistic (TSS). Two omissions (total and average) and commission (total and average) measures to test model performance were calculated [36]. The equal sensitivity and specificity threshold was used for the TSS calculation. TSS can be defined as sensitivity + specificity– 1 and values range from 0 to 1. A value of > 0.6 is considered ‘good’ and > 0.8 as ‘excellent’ [37]. Likewise, a successful model will have an AUC score approaching 1.0 and a model predicting no better than random will have an AUC approaching 0.5 [1, 38]. AUC analysis is independent of both threshold setting and prevalence, making it a very effective method for model evaluation when working with presence-only data [36]. Araújo and Guisan [38] defined a rough guide for classifying models based on their accuracy (AUC): 0.6–0.7 poor, 0.7–0.8 average, 0.8–0.9 good, 0.9–1 excellent. AUC analysis is independent of both threshold setting and prevalence, making it a very effective method for model evaluation when working with presence-only data [36]. A threshold of 80% was used to identify the area as suitable for the occurrence of anthrax in the environment. This threshold was chosen based upon the assumption that areas with a very high probability (>80%) of anthrax occurrence have the potential to be ecologically suitable for harbouring spores, thus anthrax is endemic. Areas with a lower probability of anthrax occurrence are most likely propagating epidemic occurrences.

## Results

Twelve environmental variables were retained to construct the final Maxent model (Table 1, S2 Fig). On average, model convergence was reached after 2700 iterations. The average AUC score for the model was 0.9, which was significantly different from a line of no information (p < 0.001). The model performed very well on the test data with an average AUC of 0.8. The TSS indicated a moderate performance of the model (TSS = 0.6), based on the average of 10 partitions and the defined threshold of 1.0. However, this result still differs significantly from chance (TSS = 0). The model had a total omission of 25.8% and an average omission of 23.5%. Potentially suitable habitat was predicted with a high success rate of 79.8% for the equal sensitivity-specificity threshold and was statistically significant (p < 0.001). All accuracy metrics are summarized in Table 2.

**Table 1:** Environmental variables used for the final Maxent model.

Variable ^a^	Type of data	Source	References
Integrated NDVI (indvi)^**b**^	Reflectance derived	MODIS-TERRA	[39]
Elevation (altitude)	Elevation derived	Aster-DEM	[40]
Distance to permanent water (permdist)	Distance metrics	ArcGIS Spatial Analyst extension	[29]
Distance to seasonal water (seasdist)	Distance metrics	ArcGIS Spatial Analyst extension	[29]
Distance to ephemeral water (ephdist)	Distance metrics	ArcGIS Spatial Analyst extension	[29]
Geology (geologyventer)^c^	Soils	KNP Scientific Services Skukuza	[25, 26, 41]
SOTER Soil ID (sotersoilid)	Soils	SOTER database	[27, 28]
Land Type (ltypeventer)	Soils	KNP Scientific Services Skukuza	[26]
Landscape (landscapegert)	Soils	KNP Scientific Services Skukuza	[26]
Calcium (caventer)	Soils	Interpolated from Venter database	[25, 27, 28, 42]
Precipitation of driest quarter (preqdryq)^d^	Climate	Worldclim	[24]
Temperature seasonality (tempseasonality)	Climate	Worldclim	[24]

^a^ Variable name as used in model included in parenthesis

^b^ Measure of overall productivity and biomass as derived through the small seasonal integral of time-integrated NDVI images between 2000–2009 and is not extracted for each case’s time of death.

^c^ Venter [25] classified the soils of Kruger National Park into 15 major classes and described the chemical composition of each.

^d^ Precipitation is the 50 year average 1950–2000 from the Worldclim dataset.

**Table 2:** Accuracy metrics for the predicted distribution for Maxtent model using 12 variables.

Metric^a^	Model specifications
Overall accuracy	79.5%
*N* to build models	165
*N* to test models	54
Total omission	25.8%
Average omission	23.5%
Total commission	79.8%
Average commission	79.5%
TSS	0.562
AUC	0.857

^a^ *N* indicates the number of positive point occurrences and was divided into 25% testing:75% training locations at each model subset

TSS = true skill statistic; AUC = area under curve.

**Fig 1:**
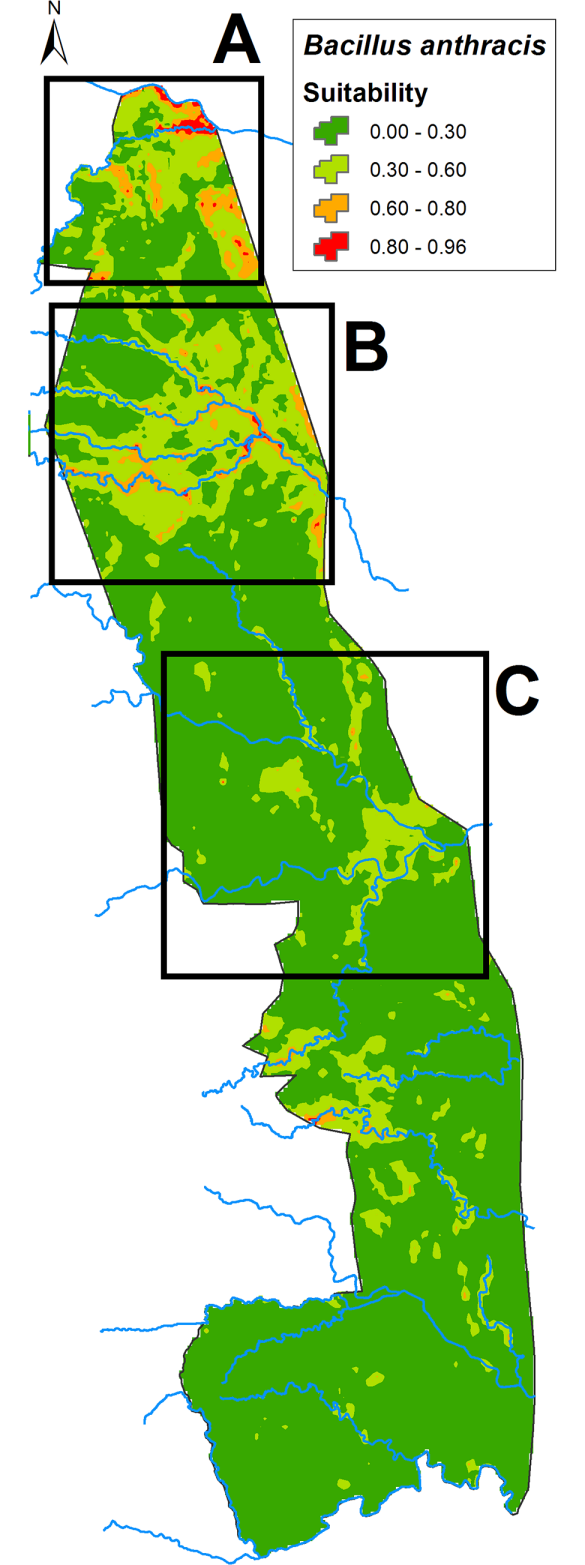
Predicted ecological suitability (>80%) for *Bacillus anthracis*. The red areas (greater than 0.8 probability) indicate areas of increased *Bacillus anthracis* ecological suitability and black rectangles indicate the three most suitable areas (A. Pafuri, B. Shingwedzi, C. Letaba) for *B*. *anthracis* detection in Kruger National Park.

Four arbitrarily defined probability classes were used to classify the ecological suitability of the modeled prediction (Table 3) ranging from high (0.8–1), moderate (0.6–0.8), low (0.3–0.6) and not suitable (0–0.3). The highly suitable area consisted of 81.67 km^2^ (0.42% of total area), 702.62 km^2^ moderate (3.61% of total area), 3943.48 km^2^ low (20.24% of total area) and 14752.23 km^2^ not suitable (75.73% of the total area). The highly suitable areas for *B*. *anthracis* (greater than 0.8) included mainly the northern Pafuri region as well as the Shingwedzi region (south of Pafuri) and the central KNP Letaba region (Fig 1).

**Table 3:** Threshold point values (>0.8) for the twelve environmental variables and the three most suitable areas identified by the model in Kruger National Park for *Bacillus anthracis* occurrence (see Fig 1).

Section	Pafuri	Shingwedzi	Letaba	Kruger National Park mean (range)
*n* pixels in area of interest	1646	3834	4447	18921
*n* cases in area of interest	302	131	81	597
*n* pixels containing cases	68	90	26	219
*n* pixels > = 0.8 threshold	75	50	4	134
Altitude (altitude)	228.806	281	225.933	292 (145–570)
Calcium (caventer)	171.835	184.626	198.049	148 (10–282)
Ephemeral Water Distance (ephdist)	2069.046	0	13181.526	3618 (0 – 20384)
Seasonal Water Distance (seasdist)	3169.587	0	7038.007	4124 (0–14764)
Permanent Water Distance (permdist)	2190.000	46840.200	733.333	12461 (0–53450)
Geology Venter (geologyventer)	LB, CS, AL	AL	EC	LB, CS, AL, EC
INDVI (indvi)	8.589	9.368	8.868	7.34 (-775–15)
Landscape (landscapegert)	25,28	35	21,22	21, 22, 25, 28, 35
Landtype (ltypeventer)	Pa04,Pa05	Le05	Le01	Le01, Le05, Pa04, Pa05
Precipitation Driest Quarter (precdryq)	9	14	23.733	14.7 (8–36)
SOTER Soil ID (sotersoilid)	ZA21,ZA22	ZA115	ZA282	ZA21, ZA22, ZA115, ZA282
Temperature Seasonality (tempseasonality)	3389.097	3687	3759.9333	3439 (2838–3828)

LB–Karoo system; Olivine rich basalts, sub-ordinate alkali basalts, shoshonites [41]. CS–Karoo system; Fine grained sandstone, mudstone, chert (Cave sandstone and redbed stages) [41]. AL–Quarternary; Alluvium [41]. EC–Karoo system; Shale with coal seams, mudstone, grit (Ecca series) [41]. 15 –*Colophospermum mopane* forest. 21 –*Combretum* / *Acacia nigrescens* rugged veld. 22 –*Combretum* / *Colophospermum mopane* rugged veld. 25 –*Adansonia digitata* / *Colophospermum mopane* rugged veld. 28 –Limpopo / Luvuvhu floodplain. 35 –*Salvadora angustifolia* floodplains. ZA21, ZA115 –Eutric cambisols (A cambisol (CM) can be defined as having either a cambic or a mollic horizon. A cambic horizon is a weakly developed mineral soil horizon and a mollic horizon is a surface horizon of mineral soil that is dark in colour, relatively deep and contains (dry weight) at least 1% organic matter or 0.6% organic carbon) [28]. Le01: Letaba landtype; Le05: Shingwedzi Landtype; includes the Shingwedzi river; Pa04 and 05: Pafuri Landtype [25]. ZA22 –Eutric leptosols (A leptosol (LP) can be defined through a limit in depth by continuous hard rock within 25 cm from the soil surface, overlying material with a calcium carbonate equivalent of more than 40 percent within 25 cm from the soil surface or less than 10 percent (by weight) fine earth to a depth of 75 cm or more from the soil surface) [28]. ZA282 –Leptic phaeozems (Continuous rock starting between 50 and 100 cm from the soil surface with a mollic horizon and (1) a base saturation of 50 percent or more and no secondary carbonates, at least to a depth of 100 cm from the soil surface and (2) with no diagnostic horizons other than an albic, argic, cambic or vertic horizon) [28].

Of the environmental variables tested (Table 1), the most important predictor in seven out of the 12 partitions was the SOTER soil class. The land type as defined by Venter [25] ranked top in the remaining five partitions. Distance to ephemeral water, soil calcium, and the integrated NDVI value ranked next, in that order, in terms of unique information, but the precipitation during the driest quarter contributed much more to the overall model gain, making this variable the third most important. Table 3 compares the values of each environmental variable used, between the range for the entire study area and the three areas in the KNP predicted to be highly suitable for *B*. *anthracis* detection.

**Fig 2:**
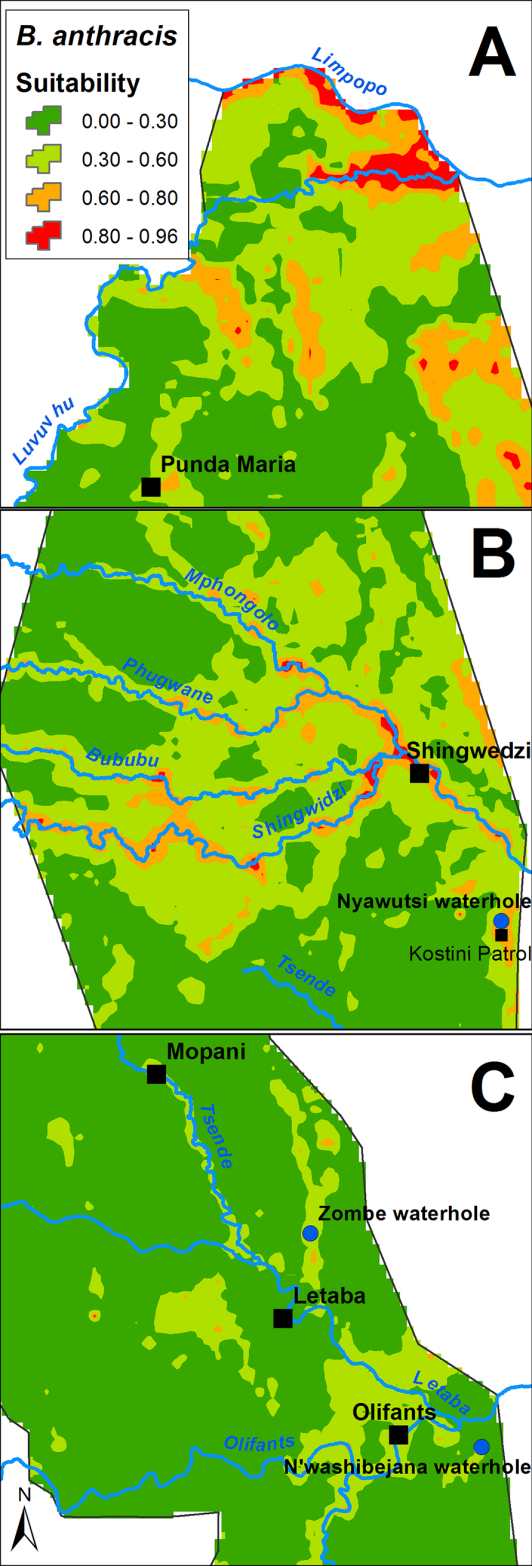
Three most ecologically suitable habitats for *Bacillus anthracis* areas in Kruger National Park. The following suitable areas for the harbouring of *B*. *anthracis* spores within the soil was identified using maximum entropy as a statistical model, a range of environmental predictors and anthrax occurrence data: (A) Pafuri in the northern Kruger National Park, (B) Shingwedzi river system and (C) Letaba / Olifants sections.

The Pafuri region in the far north of KNP is considered endemic for anthrax with periodic outbreaks, typically occurring during late winter and spreading southward [8] but since 2008 outbreaks have been recorded in the summer after rain. This endemic area lies between the Limpopo (north) and Luvuvhu (south) rivers with the most suitable areas along these river beds. A large number of drainage channels occur into this area from the higher lying southern landscape. Areas south of the Pafuri depression with high anthrax suitability (80–100%) are displayed in Fig 2A. Areas within the Shingwedzi, Mphongolo, Phugwane and Bubube rivers (Shingwedzi river system) with high anthrax suitability (80–100%) are displayed in Fig 2B. The only highly suitable area in this section that is not associated with the Shingwedzi river system is the Nyawutsi waterhole just north of the Kostini patrol camp. In the Letaba section (Fig 2C) the Zombe waterhole has a very high suitability and similarly the N'washibejana waterhole in the Olifants ranger section.

## Discussion and conclusions

This study presents the first estimate of ecologically suitable areas for anthrax spore survival in the KNP in South Africa. These ecologically suitable areas for the harboring of *B*. *anthracis* spores within the soil was identified using anthrax occurrence data, a range of environmental predictors and maximum entropy modeling. Outputs suggest there are at least three geographically distinct regions within the Park–Pafuri, Shingwedzi, and Letaba–that are highly suitable for anthrax spore survival.

Our findings of low altitude (225–280 m), high soil calcium content (171–198 me/kg), low dry quarter precipitation (9–24 mm), proximity to a water source and mineral rich, eutrophic soils as essential conditions for survival of anthrax is in agreement with most similar studies on anthrax [1, 17–19, 43].

However, the lack of influence of pH layers implicitly used in this model was surprising, although this could be due to lack of information at the spatial scale at which they were employed. Calcium (Ca) sulphate and high pH were identified as important variables in other anthrax distribution studies [1, 17, 18]. As *B*. *anthracis* is a soil borne pathogen, variables related to soil (soil moisture content, soil pH, Ca sulphate and soil type) all contributed to the potential distribution of B. *anthracis* in these studies from different continents. Ca has been shown to be important for both spore germination and the maintenance of dormancy [7]. As the soil pH increases above 7.2, due to additional soil Ca, the "free" Ca^2+^ is not absorbed into the soil and can bind with other compounds [25]. The soils of Pafuri, Shingwedzi and Letaba regions (Figs 1 and 2A–2C) are characterised by high Ca levels (and concomitant high pH), which would partly explain their high suitability for anthrax spore survival.

The importance of land [25] and soil types (SOTER), in conjunction with precipitation and NDVI [1, 17, 18] which ranked high in most model predictions, compliments the recent finding that infected carcass sites play an important role in spore dissemination to grazers, and that grazing (not drinking) seems to play a significant role at infected carcass sites [12, 13]. Altitude was not identified to play a major role in this study, which is likely due to the small variation in altitude within the KNP compared to variation at, for example, a continental scale [1, 19]. Anthrax outbreaks within KNP seem to be regulated by rainfall and the hydrology of the area [10]. The disease typically occurs during late winter when the environment is dry and animals congregate around water sources. However since 2010 outbreaks have been observed in the wet and dry season (personal communications, Skukuza State Veterinary office). The dry quarter precipitation variable of the model had an average value of 9 mm for Pafuri, 14 mm for Shingwedzi and 23 mm for Letaba. This suggests that dry environmental conditions are important for anthrax occurrence in KNP. As soon as the first rain occurs, the incidence of new cases starts to decrease. This is likely due to the washing away of spores and an increase in water sources leading to animal dispersal and alternate vulture bathing sites [8, 11]. Spore suitability appears to be associated with rivers, with apparent accumulation downstream (eastward) and peaking at confluences. This is most likely due to the alluvial deposition of spores after flooding. Spores have a high surface hydrophobicity, allowing clumping in water and a high buoyant density, allowing clumped organic matter to float [6] and settle in the soil, as water levels recede. No quantitative data supports this apparent association and should be investigated.

The relocation of *B*. *anthracis* spores with soil through water, flooding or rain could result in a new focality [9]. Most anthrax cases in Etosha National Park (ENP) in Namibia are observed in the wet season while elephants have an anthrax mortality peak in the dry season [44]. There are numerous suppositions regarding the timing of anthrax outbreaks in endemic areas such as changes in the environment or changes in animal behaviour or variation on concentration of *B*. *anthracis* in the environment. Investigating the influence of biotic and abiotic factors on the *B*. *anthracis* is complex due to the acute nature of the disease and number of variables involve in each outbreak and ecosystem.

The northern Pafuri anthrax endemic area is located at an altitude of around 228 m with an average rainfall of 400 mm per year. This area is classified by Venter [25] as the Pafuri Land Type (Pa05) and its endemicity to anthrax has previously been described by De Vos [11]. There are many small pans in the area, which during late winter usually dry up, thus creating an ideal situation for the start of an anthrax cycle. A large number of drainage channels feeds into this area from the higher lying southern landscape. Pans are replenished by run-off water after heavy rains. Soil sample analyses from the Pafuri depression clearly indicated that it acts as a catchment and accumulation area for *B*. *anthracis* spores [11]. Alluvial lowlands flank the lower Luvuvhu and Limpopo rivers with sandy to deep red silt sediments. The soil depth that anthrax spores are found at is as shallow as three centimeters during dry, high-risk outbreak conditions and as deep as 15 cm during wetter, deposition periods. Deposition periods can be defined as the time when spores are washed away during heavy rains and deposited in low-lying silt beds [11]. KNP soil profiles become shallower and soil type diversity decreases towards the north [45]. The relationship of anthrax with geographic features within the northern KNP has been described by De Vos and Bryden [10] and the effect of altitude and permanent rivers are highlighted as physical barriers to the dispersal of anthrax. The Shingwedzi river system confluence is a low-lying area with an average altitude of 260 m and an average annual rainfall of 430 mm. Significant alluvial deposits occur along this river system and various drainage channels exist in this area. The river system is not permanent and will only act as a physical barrier during wet years and after heavy rains. Very high pH and Ca levels occur along valley bottoms with predominantly dense and heterogeneous riverine vegetation. The riverine areas immediately north and west of the Shingwedzi rest camp have the highest suitability ranking in this section. With the Letaba section, the proximity of the perennial Letaba and Olifants rivers makes the spread of anthrax from these areas highly unlikely. All of the high suitability areas are associated with stagnant water sources like dams, pans, and waterholes. This study confirmed the endemicity of the Pafuri region to anthrax but also identified a number of other potential endemic sites. However, it is important to note that this is only a measure of the potential of the environment to successfully harbor anthrax spores and not the actual anthrax occurrence probability.

## Supporting information

S1 Table Worldclim variables used in Maxent model. (DOCX)

S2 Table Overview of environmental data used in Maxent indicating the variables, type of data and source. (DOCX)

S1 Fig Spatially unique cases of *Bacillus anthracis* and the predicted suitability for *B*. *anthracis* occurrence. (PNG)

S2 Fig Twelve environmental variables used in the final Maxent model. (PNG)
