# Supplementary material for: Ecological suitability modeling for anthrax in the Kruger National Park, South Africa
Source: PLoS One. 2018 Jan 29;13(1):e0191704. doi: 10.1371/journal.pone.0191704 (PMC5788353; doi:10.1371/journal.pone.0191704)
Supplement: S1 Table — (DOCX) [file pone.0191704.s001.docx]

**S2 Table: Overview of environmental data used in Maxent indicating the variables, type of data and source.**

| **Variable** | **Data description** | **Source** |
| --- | --- | --- |
| Integrated NDVI | Measure of overall productivity and biomass as derived through the small seasonal integral of time-integrated NDVI images between 2001-2008 | MODIS-TERRA |
| Maximum NDVI | MaxNDVI is the annual maximum NDVI value. This is another measure of overall productivity and biomass. The yearly maximum values were determined in ArcGIS and a mosaic raster was created to produce the index of overall productivity. | MODIS-TERRA |
| Elevation | Height above sea level in meters | Aster-DEM |
| Slope | The grade of a physical feature, refers to the tangent of the angle of that surface to the horizontal. Elevation derived. | DEM-derived |
| Aspect | Downslope direction of the maximum rate of change in value from each cell to its neighbours. Elevation derived. | DEM-derived |
| Distance to permanent water | Distance of specified point to water with hydrological index (HI) of 4.39-16.10. | ArcGIS Spatial Analyst extension |
| Distance to seasonal water | Distance of specified point to water with hydrological index (HI) of 6.11-37.81. | ArcGIS Spatial Analyst extension |
| Distance to ephemeral water | Distance of specified point to water with hydrological index (HI) of more than 37.81. | ArcGIS Spatial Analyst extension |
| Distance to boreholes | Distance of specified point to nearest borehole | ArcGIS Spatial Analyst extension |
| SOTER Soil ID | 0-ZW74  1-ZA21  2-ZA22  3-ZA29  4-ZA32  5-ZA41  6-MZ22  7-ZA55  8-ZA56  9-ZA62  10-ZA65  11-ZA80  12-ZA83  13-MZ223  14-ZA87  15-ZA98  16-ZA101  17-ZA115  18-ZA33  19-ZA145  20-MZ16  21-ZA160  22-ZA170  23-ZA189  24-ZA190  25-ZA202  26-ZA233  27-ZA245  28-ZA258  29-ZA262  30-ZA282  31-ZA283  32-ZA291  33-ZA314  34-ZA335  35-ZA357  36-ZA387  37-ZA450  38-ZA491  39-ZA533  40-ZA548  41-ZA564  42-ZA604  43-ZA610  44-ZA634  45-ZA641 | SOTER database |
| Land Type | BU01-Bulweni  BU02-Marithenga  BU03-Tsotsi  KL01-Gorge  KL02-Klipkoppies  LE01-Olifants  LE02-Letaba  LE03-Mooiplaas  LE04-Manyeleti  LE05-Shingwedzi  LE06-Mashikiri  LE07-Shilawuri  MA01-Malelane  MA02-Stolsnek  NW01-Nwambiya  NW02-Masokosa  PA01-Punda  PA02-Madzaringwe  PA03-Lanner gorge  PA04-Baobab hill  PA05-Pafuri  PA06-Malonga  PH01-Houtboschrand  PH02-Shindyanamani  PH03-Tsheri  PH04-Phalaborwa  PH05-Shivhulani  PH06-Malopeni  PH07-Mahlangeni  PH08-Tsende  PH09-Nalatsi  PH10-Bububu  PH11-Mphongolo  PH12-Dothole  SA01-Satara  SA02-Mavumbye  SA03-Bangu  SA04-Balule  SA05-Orpen  SA06-Salitje  SK01-Pretoriuskop  SK02-Napi  SK03-Randspruit  SK04-Lwakahle  SK05-Makhuthwanini  SK06-Renosterkoppies  SK07-Skukuza  SK08-Nhlanguleni  SK09-Muzandzeni  SK10-Rabelais  SK11-Timbavati  SP01-Sabiepoort  SP02-Rietpan  SP03-Pumbe  SP04-Nwanetsi  VU01-Vutome | KNP Scientific Services Skukuza |
| Landscape | 1-Lowveld sour bushveld of Pretoriuskop  2-Malelane mountain bushveld  3-*Combretum collinum/Combretum zeyheri* woodland  4-Thickets of the Sabie and Crocodile river  5-Mixed *Combretum/Terminalia sericea* woodland  6-*Combretum/Colophospermum mopane* woodland of Timbavati  7-Olifants river rugged veld  8-Phalaborwa sandveld  9-*Colophospermum mopane* woodland/savanna on basic soil  10-Letaba river rugged veld  11-Tsende sandveld  12-*Colophospermum mopane/Acacia nigrescens* savanna  13-*Acacia welwitschii* thickets on Karoo sediments  14-Kumana sandveld  15-*Colophospermum mopane* forest  16-Punda Maria sandveld on cave sandstone  17-*Sclerocarya birrea caffra/Acacia nigrescens* savanna  18-Dwarf *Acacia nigrescens* savanna  19-Thronveld on gabbro  20-Bangu rugged veld  21-*Combretum/Acacia nigrescens* rugged veld  22-*Combretum/Colophospermum mopane* rugged veld  23-*Colophospermum mopane* shrubveld on basalt  24- *Colophospermum mopane* shrubveld on gabbro  25-*Adansonia digitata/ Colophospermum mopane* rugged veld  26- *Colophospermum mopane* shrubveld on calcrete  27-Mixed *Combretum/Colophospermum mopane* woodland  28-Limpopo/Luvuvhu floodplains  29-Lebombo south  30-Pumbe sandveld  31-Lebombo north  32-Nwambiya sandveld  33-*Pterocarpus rotundifolius/Combretum collinum* woodland  34-Punda Maria sandveld on Waterberg sandstone  35-*Salvadora angustifolia* floddplains | KNP Scientific Services Skukuza |
| Basalt or Granite | Soils | KNP Scientific Services Skukuza |
| Land Cover | Soils | KNP Scientific Services Skukuza |
| Geology | MN - Alcaline (nephelinite) lavas  AL - Alluvium  WA - Andesite with subordinate tuff  MV - Conglomerate, marl, sandstone (Malvemia beds)  DI - Diabase  CS - Fine grained sandstone, mudstone, chert  AG - Granite, gneiss, migmatite, amphibolite, schist  GY - Granophyre  OG - Olivine gabbro  SB - Olivine-poor basalts  LB - Olivine-rich basalts, subordinate alkali-basalts  RS - Red and yellow sand  R - Rhyolite, dacite  WS - Sandstone, quartzite, with intercalated andesite  EC - Shale with coal seams, mudstone, grit | KNP Scientific Services Skukuza |
| Calcium | Extracted from Venter database according to land type | Venter database |
| Lithology SOTER | MA2 - Gneiss, UF - Fluvial, SC2 - Sandstone, IB2 - Basalt, SC1 - Conglomerate, IA1 - Diorite, IA4 – Rhyolite | Extracted from SOTER database |
| pH Venter | Extracted from Venter database according to land type | Venter database |
| pH SOTER | Soils | Extracted from SOTER database |
| Cation Exchange Capacity (CEC) | Soils | Venter database |
| Total Available Water Capacity (TAWC) | Soils | Extracted from SOTER database |
| Soil Clay % | Soils | Extracted from SOTER database |
| Soil Silt % | Soils | Extracted from SOTER database |
| Soil Sand % | Soils | Extracted from SOTER database |
| Annual mean temperature | Annual trend. The bioclimatic variables represent annual trends (e.g., mean annual temperature, annual precipitation) seasonality (e.g., annual range in temperature and precipitation) and extreme or limiting environmental factors (e.g., temperature of the coldest and warmest month, and precipitation of the wet and dry quarters; a quarter is a period of three months). Data from 50 year average 1950-2000. | WorldClim |
| Annual precipitation | 50 year average 1950-2000 | WorldClim |
| Isothermality | 50 year average 1950-2000 | WorldClim |
| Maximum temperature warmest month | 50 year average 1950-2000 | WorldClim |
| Mean diurnal range | 50 year average 1950-2000 | WorldClim |
| Mean temperature warm quarter | 50 year average 1950-2000 | WorldClim |
| Mean temperature wet quarter | 50 year average 1950-2000 | WorldClim |
| Mean temperature dry quarter | 50 year average 1950-2000 | WorldClim |
| Minimum temperature of coldest month | 50 year average 1950-2000 | WorldClim |
| Precipitation of driest month | 50 year average 1950-2000 | WorldClim |
| Precipitation of driest quarter | 50 year average 1950-2000 | WorldClim |
| Precipitation seasonality | 50 year average 1950-2000 | WorldClim |
| Precipitation of wettest month | 50 year average 1950-2000 | WorldClim |
| Precipitation of wettest quarter | 50 year average 1950-2000 | WorldClim |
| Temperature annual range | 50 year average 1950-2000 | WorldClim |
| Temperature seasonality | 50 year average 1950-2000 | WorldClim |
