# Supplementary figures and images for: Ecological suitability modeling for anthrax in the Kruger National Park, South Africa
Source: PLoS One. 2018 Jan 29;13(1):e0191704. doi: 10.1371/journal.pone.0191704 (PMC5788353; doi:10.1371/journal.pone.0191704)

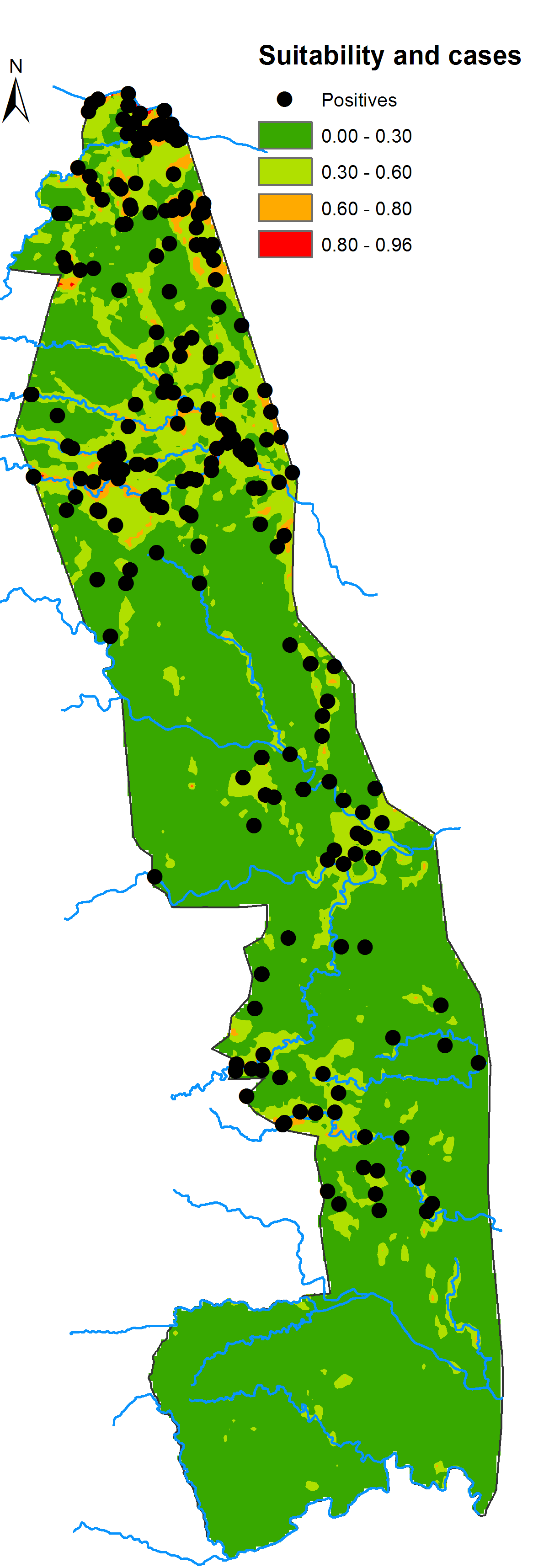

Supplement: S1 Fig — (PNG) [file pone.0191704.s003.png]

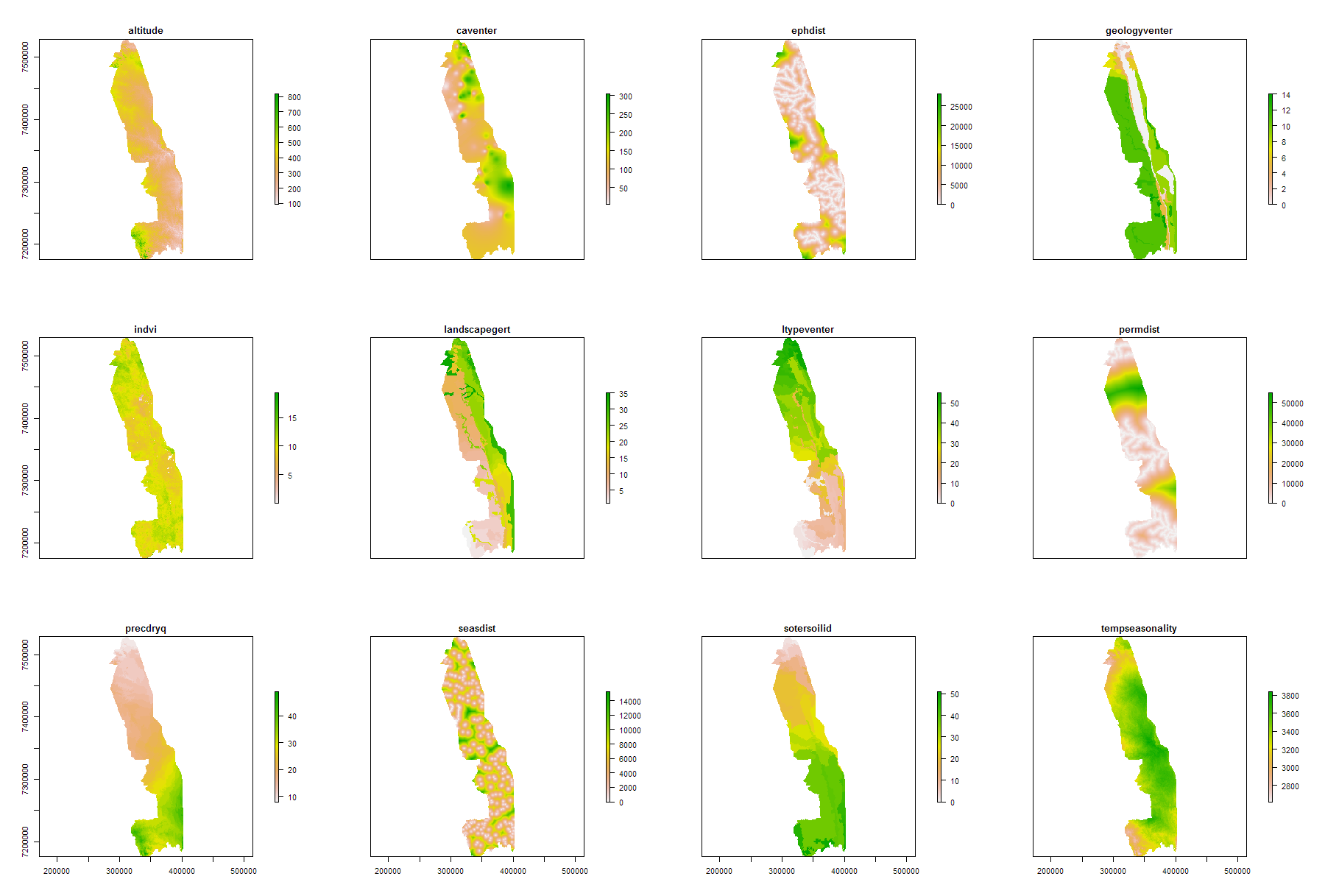

Supplement: S2 Fig — (PNG) [file pone.0191704.s004.png]
